# The Role of Inflammatory Mediators in the Pathogenesis of Obesity

**DOI:** 10.3390/nu16172822

**Published:** 2024-08-23

**Authors:** Estera Bakinowska, Mariusz Krompiewski, Dominika Boboryko, Kajetan Kiełbowski, Andrzej Pawlik

**Affiliations:** Department of Physiology, Pomeranian Medical University, 70-111 Szczecin, Poland; esterabakinowska@gmail.com (E.B.); mariusz.krompiewski@gmail.com (M.K.); dominikaboboryko@gmail.com (D.B.); kajetan.kielbowski@onet.pl (K.K.)

**Keywords:** obesity, inflammation, cytokines, chemokines

## Abstract

Obesity is a pandemic of the 21st century, and the prevalence of this metabolic condition has enormously increased over the past few decades. Obesity is associated with a number of comorbidities and complications, such as diabetes and cardiovascular disorders, which can be associated with severe and fatal outcomes. Adipose tissue is an endocrine organ that secretes numerous molecules and proteins that are capable of modifying immune responses. The progression of obesity is associated with adipose tissue dysfunction, which is characterised by enhanced inflammation and apoptosis. Increased fat-tissue mass is associated with the dysregulated secretion of substances by adipocytes, which leads to metabolic alterations. Importantly, the adipose tissue contains immune cells, the profile of which changes with the progression of obesity. For instance, increasing fat mass enhances the presence of the pro-inflammatory variants of macrophages, major sources of tumour necrosis factor α and other inflammatory mediators that promote insulin resistance. The pathogenesis of obesity is complex, and understanding the pathophysiological mechanisms that are involved may provide novel treatment methods that could prevent the development of serious complications. The aim of this review is to discuss current evidence describing the involvement of various inflammatory mediators in the pathogenesis of obesity.

## 1. Introduction

Obesity is frequently referred to as the pandemic of the 21st century. Recent epidemiological data support the use of this term [1], as one in eight people are estimated to suffer from obesity. According to a recently published pooled analysis that included 222 million people [2], during the last three decades the prevalence of obesity has increased enormously. Therefore, obesity is a significant global burden. The main risk factors for obesity are the excessive consumption of high-energy-yield foods and a lack of physical activity. However, this metabolic disease is not limited to increased body weight and accumulated fat tissue. Obesity is strongly associated with cardiovascular diseases, type 2 diabetes, and the occurrence of cancer [3]. Furthermore, as fat tissue is an endocrine organ, adipose tissue accumulation is associated with a largely dysregulated hormone profile and altered levels of adipokines, which are hormones that are secreted by adipose tissue. Additionally, obesity is associated with systemic inflammation. Understanding the complex nature of obesity and its influence on the immune system may bring novel knowledge explaining how obesity is related to autoimmune diseases [4] and cancer [5]. It could also lead to the introduction of novel treatment methods targeting inflammatory responses that could prevent the occurrence of severe comorbidities or obesity-related complications. The aim of the present review is to discuss the involvement of inflammation in the pathogenesis of obesity. 

## 2. Brief Overview of the Inflammatory Landscape of Obesity

Adipose tissue is classified as a type of connective tissue, and its main functions include energy storage, thermoregulation, and providing structural support (e.g., mesentery). It also affects the metabolism of sex hormones (oestrogens and androgens), glucocorticoids, and vitamin D [6,7]. Importantly, adipocytes participate in innate and adaptive immunity by mediating cellular and humoral mechanisms [8,9]. White, brown, and beige types of adipose tissue can be distinguished based on their structures and specific functions [10]. Due to its highly active role in metabolic and endocrine mechanisms, adipose tissue is also considered a major endocrine organ [11]. Adipocytes secrete numerous proteins known as adipokines. These molecules regulate the metabolism and have immunoregulatory properties. These proteins include leptin, adiponectin (Acrp30, AdipoQ, and apM1), adipsin, resistin, chemerin, and the relatively recently discovered omentin, among many others. Over the years, an accumulating amount of evidence demonstrated that altered expression of adipokines is involved in the pathogenesis of a variety of diseases [12,13]. It is important to note that adipose tissue also expresses some cytokines that are expressed by other types of tissues, including TNF-α, IL-1β, progranulin (PGRN), retinol-binding protein 4 (RBP4), and lipocalin-2 (LCN2) [13]. Both insufficient amounts of adipose tissue (as in individuals with anorexia) and excessive amounts of adipose tissue (as in individuals with obesity) can modify its endocrine function. In obesity, the levels of inflammatory mediators increases, and the recruitment of immune cells through chemokine signalling pathways (including CCL2 and CCL5) contributes to the transition of inflammation into a chronic process [14]. Moreover, inflammatory cells that infiltrate adipose tissue play an enormous role in stimulating pro-inflammatory conditions. Macrophages represent major producers of tumour necrosis factor α (TNF-α), a pro-inflammatory cytokine. Adipose tissue macrophages (ATMs) represent a cellular compartment in adipose tissue that can increase in pathological conditions, such as obesity [15]. Therefore, a greater abundance of these cells is associated with enhanced inflammatory responses, which will be discussed in more detail in the following sections. Furthermore, various subtypes of T cells are also present in adipose tissue, alterations of which have been observed in obesity [16,17]. 

## 3. How Is Obesity Linked to Inflammation?

### 3.1. Inflammatory Mechanisms Surrounding Adipocytes

Inflammatory cytokines have been studied in the context of obesity for decades. To begin with, tumour necrosis factor α (TNF-α) is a pleiotropic pro-inflammatory cytokine that is implicated in the pathogenesis of obesity and its complications. In an early study performed by Gokhan et al., the authors evaluated the expression of TNF-α mRNA in adipose tissue that was obtained through fat biopsies; TNF-α was upregulated in the adipose tissue of obese patients. Moreover, this expression was positively correlated with insulin concentrations in plasma collected from fasting individuals, suggesting the involvement of TNF-α in insulin resistance, which is frequently associated with obesity. Importantly, weight reduction could reduce the expression of TNF-α in adipose tissue [18]. Furthermore, higher levels of TNF-α were detected in blood from obese patients [19], and these levels were further increased in blood from obese patients with T2DM [20]. Genetic polymorphisms of the TNF-α gene were found to be significantly associated with the risk of obesity in certain populations, further confirming the association with the condition [21,22].

Therefore, it is important to discuss the influence that TNF-α has on adipocyte pathophysiology during the progression of obesity. Overconsumption requires the storage of lipids, which largely occurs in adipose tissue. Hypertrophy and hyperplasia of adipocytes allow them to store large amounts of triglycerides. Eventually, hypertrophy leads to several pathophysiological events, including endoplasmic reticulum stress (ERS) or the excess generation of reactive oxygen species (ROS), that induce adipocyte dysfunction [23]. Regarding the ERS, as increased intake of calories requires cellular storage, the ER needs to synthesise an accumulating number of proteins that would allow to create lipid droplets. Moreover, an excessive amount of lipid metabolites affect ER sensors, which is associated with ERS [24]. Weight gain is also associated with adipocyte apoptosis, as demonstrated by increased activity of caspase-3, which is an effector of programmed cell death [25]. Dose-dependent stimulation of adipocytes with TNF-α increases the expression of caspases and also enhances adipocyte apoptosis. The pro-apoptotic effect of the TNF-α cytokine was attenuated by ghrelin, which is a peptide with pro-adipogenic properties [20]. Furthermore, the knocking out of adipocyte caspase-8 in mice was associated with less weight gain, together with reduced inflammation of the adipose tissue [26], confirming the involvement of caspases in the pathogenesis of obesity and inflammation. 

**Figure 1:**
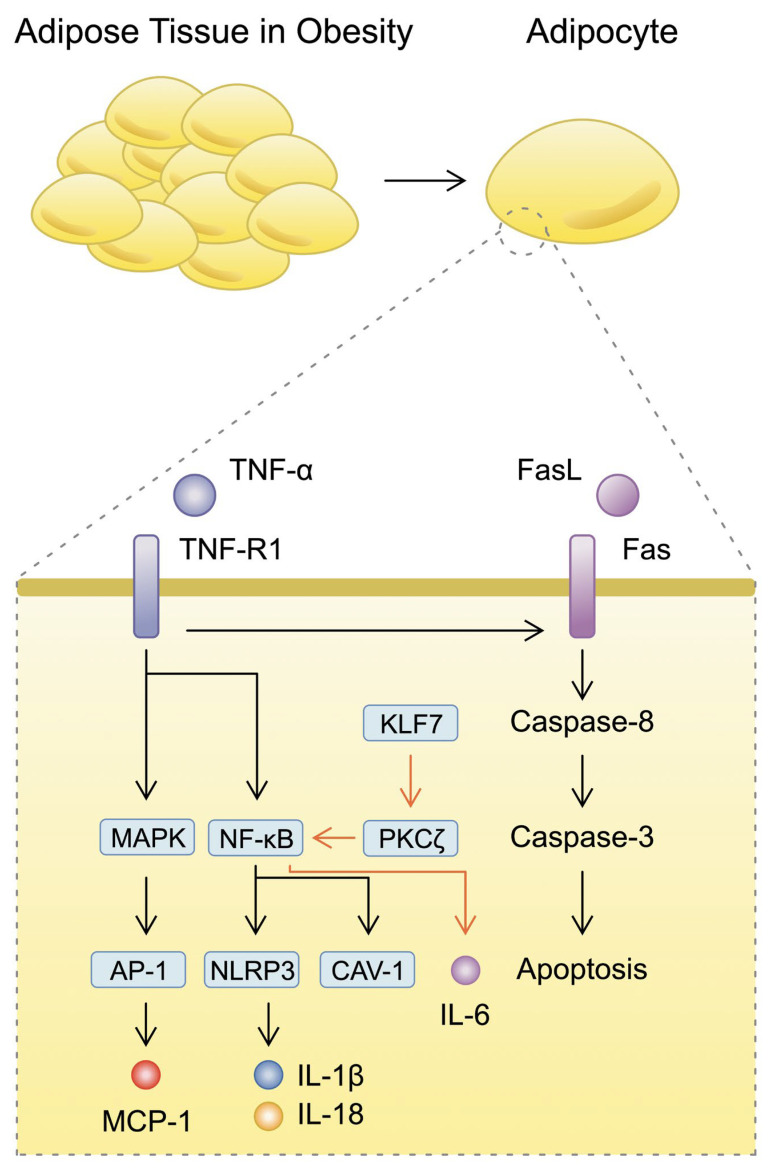
A schematic illustration demonstrating pro-inflammatory and pro-apoptotic pathways present in adipose tissue of patients with obesity.

To exert its functions, TNF-α binds to and signals through its receptors, TNF-R1 and TNF-R2. Downstream signalling through these receptors requires adaptor proteins and ultimately activates various signalling pathways, including a major pro-inflammatory transcription factor known as nuclear factor kappa B (NF-κB) [27]. In addition, TNF-α mediates the activation of the mitogen-activated protein kinase pathway (MAPK) and also activator protein-1 (AP-1). In adipocytes, TNF-α was found to induce the expression of monocyte chemoattractant protein 1 (MCP-1), which is a chemokine that stimulates monocyte accumulation in adipose tissue, and it will be described further below [28]. The state of obesity is also associated with higher expression of the pro-apoptotic receptor Fas [29]. Figure 1 depicts pathways and molecules that are induced by TNF-α in adipocytes. Activation of NF-κB leads to enhanced pro-inflammatory responses and enhanced immune cell migration and infiltration of adipose tissue [30]. Importantly, this transcription factor has been found to take part in the pathogenesis of numerous diseases, including obesity. Specifically, the TNF-α/NF-κB pathway regulates the expression of caveolin-1 (CAV-1); this is a functional and structural protein of caveolae, which are cholesterol-rich lipid rafts present in the plasma membrane. Elevated expression of CAV-1 is observed in subcutaneous adipose tissue obtained from obese patients. Furthermore, its expression is positively correlated with the expression of both TNF-α and NF-κB. Stimulation of adipocytes with TNF-α was shown to increase the expression of CAV-1 [31]. This is an important finding that identifies the upstream regulators of CAV-1; silencing of these regulators is associated with resistance to obesity, but also with metabolic alterations in animal models [32]. In epididymal white adipose tissue from mice fed with a high-fat diet, increased expression of elements of the NF-κB, as well as that of Krüppel-like factor 7 (KLF7) and protein kinase Cζ (PKCζ) was found. NF-κB is implicated in an interaction network with several signalling cascades that regulate inflammatory responses. According to Yang et al., the KLF7/PKCζ/NF-κB axis is associated with elevated expression of IL-6 and impaired glucose consumption in 3T3-L1 adipocytes [33]. Nevertheless, the roles played by NF-κB and the molecules that regulate its activity seem to be more complex. In an inflammatory context, molecules and signalling cascades are frequently found to induce both beneficial and harmful effects. Similar findings have been observed regarding the transcription factor NF-κB. Specifically, IkB kinase β (IKKβ) enhances the activity of NF-κB, and thus it is associated with the inflammatory response. However, Park and colleagues found that IKKβ silencing did not affect weight gain but did stimulate insulin resistance. Furthermore, the authors demonstrated that it protected adipocytes from death and from alterations induced by a high-fat diet [34].

Interestingly, the impact of obesity seems to spread across the adipose tissue and induce pro-inflammatory responses in surrounding adipocytes. Specifically, stimulation of 3T3-L1 adipocytes with conditioned medium obtained from obese adipose tissue stimulated the NF-κB pathway and ROS generation. Moreover, stimulation significantly increased the secretion of pro-inflammatory mediators such as IL-6, TNF-α, and IL-1β [35]. 

Overall, numerous inflammatory mechanisms surround adipose tissue which is associated with chronic systemic inflammation. Obesity is associated with increased activity of inflammatory signalling pathways that regulate the secretion of pro-inflammatory mediators and chemokines, as well as adipocyte apoptosis. Importantly, weight loss is associated with suppressed systemic inflammation, including the expression of NF-κB and levels of circulating TNF-α [36]. Apart from lifestyle modification, agents that target and suppress pro-inflammatory responses could be associated with beneficial effects in patients with obesity. Canakinumab, an agent targeting IL-1β, improves glycaemic control and reduces levels of pro-inflammatory mediators in patients with T2DM and COVID-19 infection [37]. Perhaps, targeting IL-1β could be used in patients with obesity to suppress the development of diabetes in the future. Current evidence also suggests that agents targeting TNF-α could provide important benefits. Carvalho et al. showed that treatment of obese Wistar rats with trypsin inhibitor from tamarind seeds (TTI) reduced food intake and improved the lipid profile of these animals [38]. Interestingly, the agent could also reduce plasma levels of leptin [39], a hormone released from adipocytes. In obesity, elevated levels of leptin are frequently observed, which is known as leptin resistance. As the adipokine has pro-inflammatory effects, its elevated concentrations could be associated with systemic inflammation [40]. 

### 3.2. Immune Cells in Obesity

The infiltration of immune cells into adipose tissue is characterised by high diversity and sensitivity to processes related to obesity and ageing [41,42]. The amount of visceral adipose tissue (VAT) increases in obesity, and this increased distribution leads to the development of metabolic dysregulation. Macrophages are by far the most numerous type of immune cells in adipose tissue [43].

Macrophages are present in nearly all human tissues and they perform tissue-specific functions, thereby also fulfilling immune and cleansing roles as mononuclear phagocytes [44,45]. They also serve as a source of pro-inflammatory molecules, and they indirectly affect the production of acute-phase proteins. In a 2003 study, Weisberg et al. demonstrated that the number of adipose tissue macrophages increases in obesity, participating in activated inflammatory pathways; the researchers proved that adipose tissue macrophages (ATM) in obese individuals were responsible for significant proportions of the expression of inducible nitric oxide synthase (iNOS) and IL-6; most importantly, ATM were responsible for nearly all of the expression of TNF-α [46]. 

Based on expression patterns and gene functions, two main phenotypes of macrophages can be distinguished: classically activated macrophages (M1) and alternatively activated macrophages (M2) [47]. M1 polarisation occurs in response to signals from the IFN-γ and lipopolysaccharide (LPS) pathways and leads to the production of various pro-inflammatory chemokines, including CXCL1, CCL5, CXCL16, and CXCL9; this polarisation subsequently mobilises Th1, Tc1, and NK cells [48]. Anti-inflammatory M2 cells, unlike M1 cells, are characterised by low levels of production of pro-inflammatory cytokines such as IL-1, TNF, and IL-6. M2 cells participate in tissue regeneration and also promote Th2-type immunity. Their polarisation leads to the production of CCL1, CCL18, CXCL13, CCL17, and CCL24. It is recognised that the expression of M1 chemokines is inhibited by signals inducing M2 polarisation, including IL-10, IL-4, and IL-13 [48]. Depending on the specific stimuli regulating M2 polarisation, they can be subdivided into M2a, M2b, M2c, and M2d subtypes [49]. 

Obesity caused by excessive energy intake leads to a shift in the activation state of ATMs from the anti-inflammatory M2 to the pro-inflammatory M1, thereby promoting chronic inflammation and contributing to the development of insulin resistance and type 2 diabetes [47,50]. Additionally, in 2014, Kratz et al. demonstrated that high levels of exposure to glucose, insulin, and palmitate can lead to the metabolic activation of macrophages (MMe), resulting in the formation of a unique phenotype of pro-inflammatory macrophages with surface marker expression that is distinct from that of M1s [51]. During inflammation, macrophages also play roles in antigen presentation and in the activation of various subtypes of T lymphocytes [52]. 

**Figure 2:**
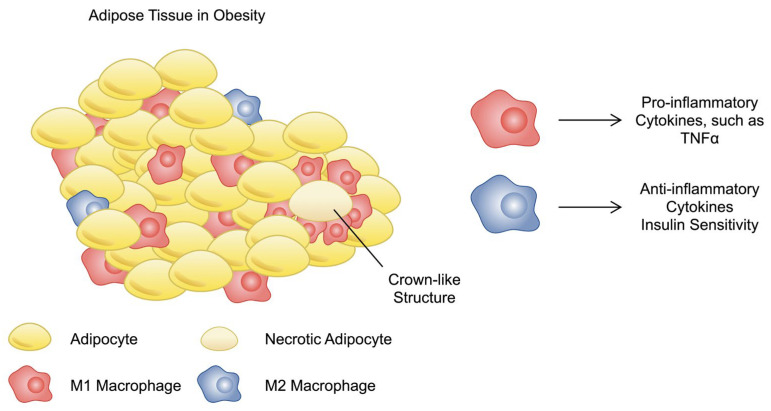
A schematic presentation of adipose tissue in obesity. The tissue is infiltrated with pro-inflammatory macrophages which secrete inflammatory mediators, such as TNF-α. M2 macrophages play a protective role by secreting anti-inflammatory cytokines and enhancing insulin sensitivity.

The presence of the anti-inflammatory phenotype of macrophages is crucial to suppress pathophysiological mechanisms associated with obesity. The M2 variants secrete exosomes containing microRNA (miRNA)-690 to stimulate insulin sensitivity. miRNAs belong to the broad family of non-coding RNAs (ncRNAs), members of which are major regulators of gene expression. Cells can secrete ncRNAs in exosomes to affect the behaviour of other cells. The classic mechanism of action of miRNAs involves them binding to their target mRNA to suppress translation, which significantly affects cellular responses and the activity of signalling pathways. miR-690 was found to mediate insulin sensitivity by binding to Nadk, which regulates glucose uptake and insulin signalling [53]. M2 macrophages induce a beneficial metabolic effect, which opposes the one induced by obesity. Crucial in intercellular signalling, macrophage-derived exosome cargo can be modulated. Specifically, stimulation of macrophages alters ncRNA molecules present in extracellular vesicles. Exosomes obtained from IL-4 stimulated cells could enhance the presence of anti-inflammatory macrophages. In obese mice models, introduction of exosomes derived from IL-4 stimulated cells changed the profile of immune cells and reduced cardiometabolic inflammation [54]. Importantly, researchers have suggested that modification of the macrophage phenotype might not require stimulation with exosomes or cytokines. Using mice models, Lin and colleagues proved that exercise enhances the presence of M2 macrophages in adipose tissue. Mechanistically, exercise reduced the expression of parvalbumin, a negative regulator of the anti-inflammatory macrophage variant [55]. Another potential mechanism that induces the presence of M2 macrophages could involve irisin. It is an exercise-dependent myokine, which enhances the polarisation of anti-inflammatory variants of macrophages, which was recently demonstrated by Tu and colleagues [56]. Additionally, a combination of exercise with dietary restriction was also associated with the anti-inflammatory macrophage population [57]. Recently, other researchers have shown the beneficial anti-inflammatory effects of exercise or its combination with other methods [58,59]. Therefore, taking into consideration the role of inflammatory molecules and signalling in obesity and obesity-related comorbidities, as well as beneficial functions of agents that target inflammatory mediators, strategies to modulate macrophage polarisation could result in beneficial outcomes as well. Figure 2 describes the involvement of macrophages in adipose tissue. Importantly, there are other subtypes of macrophages than the classic M1 and M2 subtypes [60,61]. Therefore, future studies should try to examine the involvement of particular subvariants in the adipose tissue of patients with obesity.

### 3.3. Chemokines

Chemokines play key roles in the migration and distribution of all immune cells. They bind to G protein-coupled receptors on target cells [62,63] and they are involved in organ development, angiogenesis, haematopoiesis, tumorigenesis, and cancer metastasis [64,65]. There are four main types of chemokines: CC, XC, CXC, and CX3C. This classification is based on the location of cysteine residues. Each family has its own receptor, including the following CCR, XCR, CXCR, and CX3C [62]. They are categorised into inflammatory and homeostatic groups. 

CCL2—also known as MCP-1—is the first-discovered and most-studied chemokine in humans [66]. CCL2 binds to its receptor, CCR2, to activate signalling pathways that are responsible for the migration of inflammatory cells [67]. It also contributes to many pathological conditions, such as cardiovascular diseases, brain pathologies, bone and joint diseases, respiratory infections, cancer, and endothelial dysfunction [68]. In obesity, there is a chronic low-grade inflammation that is associated with insulin resistance and the infiltration of adipose tissue by macrophages, for which MCP-1 is one of the main chemoattractants [69,70]. MCP-1 levels were shown to be significantly elevated in obese mice compared with those with normal body weight [71,72,73,74]; this relationship has also been confirmed by human studies that investigated obese adults [70,75,76,77] and children [78]. Higher levels of MCP-1 have also been observed in children with T1DM [79], although another study appears to suggest that MCP-1 is not elevated in children with T1DM but is elevated in obese children with coexisting T1DM [80]. MCP-1 is significantly expressed when induced by insulin. High levels of MCP-1 have been shown to reduce insulin-stimulated glucose uptake by differentiated adipocytes and also to reduce mRNA levels for lipoprotein lipase (LpL), adipsin, GLUT-4, aP2, β3-adrenergic receptor, and peroxisome proliferator-activated receptor (PPAR-γ), suggesting it is a potential factor in the development of insulin resistance [72]. In addition, MCP-1 contributes to macrophage accumulation in adipose tissue that constitutes chronic inflammation [74]. During obesity, there is an increase in pro-inflammatory M1 macrophages that is correlated with adipose tissue inflammation and insulin resistance [81,82]. One study found that it is obesity-induced insulin resistance that produces the MCP-1 protein, which is the factor that recruits monocytes and activates pro-inflammatory macrophages in adipose tissue [83]. Furthermore, this study found that it is insulin resistance that leads to inflammation in adipose tissue, rather than other way around. The higher the expression of the CCL2 gene or its receptor CCR2, the greater the likelihood of atherosclerosis development in genetically modified animals. In contrast, the deletion of CCL2 or its receptor (CCR2) in mice is followed by a significant reduction in the development of atherosclerotic plaques and obesity [84]. Interestingly, according to Sukumar et al., elevated MCP-1 levels have been shown to occur in obese individuals only when elevated parathormone (PTH) levels coexist [85]. In another study, significantly fewer successful in vitro fertilisation episodes were observed in women with obesity and elevated MCP-1 levels [76]. The results of studies evaluating the effect of physical activity on MCP-1 levels are conflicting, with some studies showing a significant increase in MCP-1 levels from physical activity [86,87] and others showing a decrease in MCP-1 levels [88,89,90]. This appears to be related to the type of physical activity in which muscle damage occurs (resistance exercise) and the immune response secondary to it related to monocyte mobilisation [86].

Compounds such as metformin and thiazolidinediones cause a decrease in MCP-1 levels, which suggests that these anti-diabetic compounds have anti-inflammatory properties in adipose tissue [91,92]. Patients treated with metformin and insulin had low serum levels of MCP-1 and cathepsin D, which suggests that this drug combination may be effective in reducing the progression of diabetic retinopathy [93]. Additionally, metformin inhibited the formation of urine crystals, possibly through downregulating the inflammatory mediators OPN (ostepontin) and MCP-1 [94]. Metformin reduces inflammatory responses by inhibiting MCP-1 without affecting renal function, which contributes to its beneficial effects in the treatment of T2DM [95]. Lisinopril—a commonly used angiotensin-converting enzyme inhibitor (ACEI)—significantly reduces MCP-1 levels in the urine of patients with diabetic nephropathy by reducing MCP-1 expression in renal tubular epithelial cells, leading to reduced monocyte migration and improved renal function [96]. Furthermore, a significant decrease in MCP-1 levels and reduced monocyte adhesion to the vascular endothelium is observed in users of GLP-1 receptor agonists (semaglutide, dulaglutide), which are drugs registered for the treatment of T2DM and obesity, thus suggesting a potential mechanism for reducing cardiovascular risk [97]. Weight-loss patients undergoing bariatric surgery show reduced MCP-1 levels [77]. Similarly, a decrease in macrophage infiltration in adipose tissue and a decrease in MCP-1 levels were observed in subjects after weight loss induced by gastric bypass surgery [98].

Recently, a relationship between MCP-1 and crown-like structures (CLSs) has been observed. CLSs are structures formed by macrophages surrounding dead adipocytes, which can indicate inflammatory conditions. Depending on the subtype of macrophages in CLSs, the presence of these structures can affect survival in cancer patients [99]. Furthermore, studies began to investigate whether there is a relationship between CLSs and cardiovascular diseases [100]. Few studies examined the role of CLSs in obesity. In a cohort of breast cancer patients, the presence of CD68+CLSs was positively associated with leptin, insulin and BMI [101]. Researchers recently demonstrated a relationship between CLSs and MCP. In mice, the expression of MCP colocalised with CLSs; and the presence of the latter structures was increased in obese animals compared to the lean ones. Interestingly, the presence and potential involvement in inflammatory responses of CLSs seem to depend on the metabolic condition. Exercise was associated with reduced abundancy of CLSs in obese animals, but with greater presence in lean mice. Furthermore, CLSs were found to colocalise with M2 macrophages, thus suggesting the involvement of these structures with the MCP chemokine and macrophages [102]. Future studies should investigate the precise interaction between CLSs with pro- and anti-inflammatory macrophages. Interestingly, CLSs could become biomarkers of metabolic disorders. For instance, in a recent publication by Fan et al., the authors analysed liver biopsies of patients with obesity and demonstrated that severity of neutrophilic CLSs is associated with the presence of metabolic dysfunction-associated steatohepatitis (MASH) [103].

CCL5, also known as RANTES, has been associated with T2DM and impaired glucose tolerance [104,105]. It is expressed by fibroblasts, platelets, macrophages, and eosinophiles. CCL5 plays a role in the recruitment of T cells, NK cells, and macrophages [106]. It has been reported that this chemokine is regulated by NF-kB [107]. Adipose tissue serves as a deposit of CCL5. Interestingly, some studies indicated that abdominal tissue remains a greater source of CCL5 than subcutaneous adipose tissue [108,109]. It was reported that CCL5 expression was increased in adipocytes with greater size [110]. A recent study demonstrated that its expression is elevated in obese humans and mice which contributes to the recruitment of pro-inflammatory cells and increased inflammation in adipose tissue [111]. Physical activity was associated with reduced expression of CCL5 in adipose tissue of patients with obesity [112]. Recently, Liao et al. investigated the role of CCL5 secreted by adipose stem cells (ASCs), another major cellular component of adipose tissue. In obesity, ASCs induce pro-inflammatory responses. By secreting CCL5, these cells significantly contribute to the migration of T cells [113]. CCL5 was previously found to be secreted by macrophages in obesity-associated adipose tissue [114]. 

Levels of CLCL9, CXCL10, and CXCL11 are significantly correlated with BMI, elevated inflammatory markers, and levels of other pro-inflammatory chemokines [115]. It has been shown that in obesity there is an increase in the concentrations of most CXC chemokines, which are strongly associated with leukocyte recruitment [116]. Obesity, and especially comorbid T2DM, have been associated with abnormally low blood CXCL14 levels and impaired CXCL14 expression in adipose tissue [117]. 

**Figure 3:**
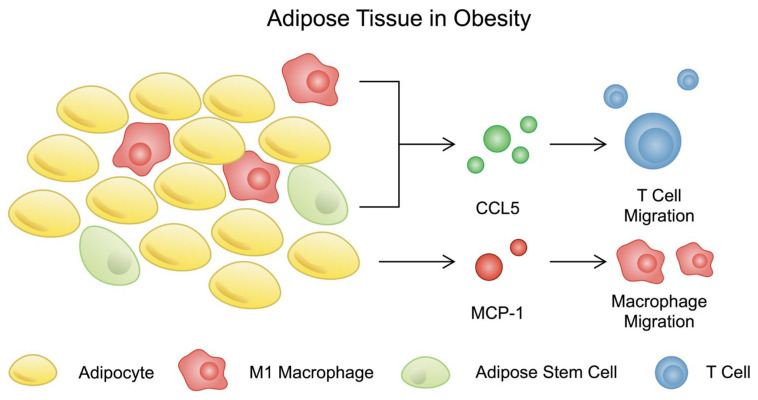
A schematic presentation of the impact of chemokines on the immune cell infiltration of adipose tissue in patients with obesity.

A better understanding of the mechanisms linking obesity and chemokines could help to identify new therapeutic targets for the treatment of obesity, which in turn could contribute to reducing the risk of cardiovascular complications, which is a major consequence of obesity. Pro-inflammatory chemokines could potentially serve as diagnostic or prognostic biomarkers in the context of obesity and its complications, and potential chemokine inhibitors (including MCP-1 or the CCR2) could represent a new class of drugs. Figure 3 summarises the role of chemokines in obesity-associated adipose tissue inflammation.

### 3.4. Inflammasome 

Another entity that is strongly associated with inflammation is the nucleotide-binding domain, leucine-rich repeat-containing family, pyrin domain-containing-3 (LRP3) inflammasome. This complex recognises damage-associated molecular patterns (DAMPs), and its activation enhances the maturation and secretion of IL-18 and IL-1β and also enhances pyroptosis. A comprehensive description of the inflammasome has been published by Swanson et al. [118]. The expression of NLRP3 is significantly greater in visceral adipocytes obtained from obese patients than in those obtained from lean people. Furthermore, NLRP3 expression is associated with a number of inflammatory cytokines, including IL-6, TNF, and IL-8, among others [119]. NLRP3 is also thought to be involved in the process of white adipose tissue browning, a mechanism that is discussed to induce beneficial effects in obesity [120]. Specifically, co-culture of mouse adipocytes with culture medium of NLRP-deficient macrophages was associated with the reduced expression of uncoupled protein 1 (UCP1), which is a marker of brown adipose tissue [121]. 

The NF-κB pathway primes NLRP3 activation [122], but other signalling pathways also contribute to its stimulation. Specifically, the lack of the mammalian target of rapamycin (mTOR) significantly enhances the expression of NLRP3 components and stimulates adipocyte inflammation [123]. Furthermore, the analysis of fat subcutaneous tissue demonstrated that the consumption of a high-fat diet induced the expression of fibroblast growth factor 2 (FGF2), which is another upstream molecule that enhances NLRP3 expression [124].

Several studies have demonstrated that targeting NLRP3 expression is a potential method for suppressing obesity-related inflammation. For instance, Liu and colleagues showed that melatonin inhibited adipocyte pyroptosis by suppressing NLRP3 activation [125]. Moreover, treatment of TNF-stimulated adipocytes with NaB, a histone deacetylase inhibitor, resulted in suppressed expression of pro-inflammatory cytokines in adipose tissue, together with suppressed immune cell infiltration and suppressed NLRP3 activation [126]. Interestingly, omega-3 fatty acids have also been shown to suppress inflammatory markers and NLRP3-associated elements, but they did not significantly affect the expression of NLRP3 itself [127]. Recently, it has been shown that the use of NLRP3 inhibitors in a mouse model of diet-induced obesity could significantly reduce body weight [128], thus demonstrating the promising potential of targeting NLRP3. Conversely, it is also important to identify drugs and compounds that stimulate the activity of the inflammasome and promote inflammation in adipose tissue. Henriksbo and collaborators suggested that fluvastatin could dysregulate NLRP3 activity in fat tissue [129]; using LPS-primed white adipose tissue explants, the authors demonstrated that fluvastatin enhanced the activity of the inflammasome.

IL-18 also known as IFN-γ-Inducing Factor (IGIF) is a pleotropic pro-inflammatory cytokine that belongs to the IL-1 family [130,131,132]. It is produced by monocytes/macrophages, dendric cells, keratinocytes, and mesenchymal cells [133]. IL-18 is produced as inactive precursor which is transformed to its active form by caspase 1 which is a part of an inflammasome. IL-18 enhances TNF-α synthesis and Th1 responses which increase INF-γ production [134]. IL-18 levels are elevated in patients with obesity [135,136]. This pro-inflammatory cytokine is related to insulin resistance and metabolic syndrome [137,138,139]. It was demonstrated that patients with obesity had increased levels of IL-18 compared to healthy controls. The highest levels of IL-18 were measured in patients with diabetes and obesity [140]. Ahmad et al. reported that IL-18R/IL-18 induced gene overexpression of different inflammatory markers such as TBF- α, CD68, CD86 in adipose tissue. Moreover the study indicated that obese and overweight patients presented higher expression of IL-18R together with its ligand in adipose tissue compared to lean controls [141]. These findings may indicate that the IL-18R/IL-18 axis may be modulated by obesity. A study by Fatima et al. reported a positive correlation between IL-18 and BMI, HOMA-IR, insulin, and waist circumference. Researchers brought attention to IL-18 polymorphism which increase IL-18 levels leading to inflammation. Therefore, current evidence suggests that IL-18 is involved in development of metabolic syndrome [142]. Another study conducted on Korean women with obesity demonstrated that the–607 C/A polymorphism of IL-18 was related to higher BMI values [143]. Straczkowski and collaborators demonstrated that in obese patients there was an inverse correlation between serum levels of IL-18 and adiponectin, a protein responsible for insulin sensitivity [144]. Moreover, Pillon and colleagues suggested that elevated levels of IL-18 in obese patients might be caused by saturated fatty acids that activate pro-inflammatory responses in human monocytes. Monocytes in obese patients enhance levels of caspases. Importantly, caspase 4/5 contributed to increased levels of IL-18 release [145]. Interestingly, IL-18 levels could serve as markers of metabolic disorders associated with obesity. In children with obesity, monitoring IL-18 could help in predicting fatty livers and advanced liver steatosis [146]. Other studies evaluated IL-18 levels in patients with obesity before and after losing weight. The patients presented a significantly reduced concentration of this cytokine after reduction in body mass compared to the condition before weight loss [147,148]. Interestingly, mice models with IL-18 knockout developed obesity independently of the type of diet (high-fat or low-fat). Moreover, IL18-/- mice had reduced whole-body energy exposure which suggests that IL-18 may be associated with the basal metabolic rate. Central administration of IL-18 did not affect food intake or the metabolic rate [149]. Another study conducted on mice models with IL-18 knockout demonstrated that mice presented increased food intake, but the metabolic rate was the same in IL-18-/- mice as in the controls. Additionally, it was reported that plasma glucose and insulin serum concentration did not differ in 3-month-old mice, while 6-month olds showed significantly higher levels of these parameters [150]. This finding is in line with a recent study by Lana et al., which again confirmed IL-18 absence was associated with obesity and impaired glucose metabolism in mice [151]. In this context, it is interesting to note that knockout of caspase-1 also resulted in the development of obesity in mice [152]. Mice with normal body weight after administration of IL-18 to their brains developed cerebrovascular impairment, which may be caused by inflammation caused by this cytokine [153]. Inhibition of IL-18 may provide potential therapeutic effects for patients with obesity. Targeting IL-18 alone by using monoclonal antibodies had limited efficacy which might be caused by contribution of other cytokines involved in inflammatory process [154]. Future studies should examine IL-18 inhibitors and antagonists in obesity treatment. Notably, studies show that administration of a combination of ezetimibe and simvastatin reduced plasma levels of IL-18 in obese patients. 

Taking into account previously published studies about the inhibiting role of IL-18 in appetite and food intake [149,150,155,156,157], elevated levels of IL-18 in obesity might suggest a compensatory mechanism. Hypothetically, an increasing BMI induces IL-18 release to suppress further food intake. However, the state of obesity might induce IL-18 resistance, which manifests as increased concentrations of this cytokine. Since the organism does not react to IL-18 signals, the high levels of the cytokine might affect other mechanisms, such as inflammation.

## 4. The Omics Studies

In the previous sections, we discussed the involvement of individual or small groups of molecules that participate in the pathogenesis of obesity. However, an increasing number of studies now examine the whole set of molecules belonging to a particular biologic system, which is broadly known as the omics studies. Numerous omics technologies are being used to comprehensively examine a set of data, such as next-generation sequencing (genomics), single cell RNA sequencing (transcriptomics), and mass spectrometry (proteomics, metabolomics), among many others [158]. The combination of these studies is termed multiomics. These methods allow to deepen our knowledge about processes involved in the pathogenesis of diseases. Importantly, monitoring a set of molecules has diagnostic potential as well. Large-scale alterations can suggest subclinical changes occurring in an organism. This approach could be crucial in conditions with subclinical progression, such as atherosclerosis. Furthermore, these techniques could possibly be implemented to identify comorbidities of obesity. 

A large-scale analysis of proteins is known as proteomics. This field of research aims to identify proteins, and analyse their structure and interactions, as well as their functions. In obesity, proteomics studies have been investigating inflammatory, metabolic, and cardiovascular alterations. According to a recently published study by Liao and colleagues, the authors used the Olink Cardiometabolic Explore panel to analyse 346 proteomic markers from the plasma of metabolically healthy (MHO) and metabolically unhealthy patients with obesity (MUO), as well as non-obese people. Intriguingly, the authors found differentially expressed proteins between metabolically healthy and unhealthy patients with obesity, but this finding did not reach significance according to the false discovery rate (FDR). The overrepresentation analysis of 40 differentially expressed proteins between patients with obesity and healthy controls revealed a significantly enriched regulation of leukocyte migration, inflammatory responses, and chemotaxis [159]. Therefore, this study confirms the involvement of systemic inflammation in obesity. Furthermore, it suggests that proteomics might be implicated in differentiating MHO and MUO cohorts. A few more studies explored this issue. In 2016, Doumatey and colleagues analysed serum proteomics of MHO and MUO patients. The authors identified 56 differentially expressed proteins, and, after FDR correction, 20 differentially expressed proteins between cohorts remained. Compared to MUO patients, people in the MHO cohort showed under-expressed acute phase reactant proteins, such as CRP, complement factor-4A (C4A), and retinol-binding protein 4 (RBP4), among others. Consequently, metabolically regulated patients with obesity are suggested to have reduced inflammatory responses [160]. In addition, lipidomics and metabolomics studies also demonstrate differences between MHO and MUO. For instance, patients without metabolic disturbances showed positive associations with sphingomyelins and phosphatidylcholine ethers [161].

## 5. Obesity and Cancer—A Focus on Immune Modulation

As previously mentioned, the presence of obesity is associated with an increased risk of cancer. Moreover, obesity regulates immune responses that are associated with anti-tumour activity. In vivo experiments showed that tumours in mice with obesity tend to grow faster than those in animals receiving control diet. The state of obesity significantly changes the profile of tumour-infiltrating leukocytes. Specifically, it decreases the presence of lymphocytes. Tumour-infiltrating CD8+ cells showed reduced expression of both costimulatory and coinhibitory molecules, and the cells demonstrated reduced proliferation potential [162]. Moving further, if obesity impairs the profile of tumour-infiltrating lymphocytes, as well as their functionality, obesity could alter the efficacy of immunotherapy. This treatment strategy enhances the cytotoxic activity of immune cells by blocking programmed cell death receptor or ligand (PD-1/PD-L1). Malignant cells use the PD-1/PD-L1 axis to suppress the anticancer properties of T cells. In fact, recent studies demonstrated that obesity could affect the response to immunotherapy. For instance, the presence of obesity in patients with renal cell cancer decreased survival [163]. However, Tan et al. showed that a high BMI could enhance survival in a cohort of patients with oral tongue squamous cell cancer treated with immunotherapy in a neoadjuvant setting [164]. Hypothetically, metabolic and immune alterations in obesity could depend on the type of cancer, as well as type of treatment. Furthermore, other regulatory mechanisms might control this influence, which should be further examined. The ability of obesity to enhance both tumour formation and immunotherapy response has been observed in other studies and termed the obesity paradox [165]. Intriguingly, obesity was recently found to enhance the expression of PD-1 on tumour-associated macrophages [165].

## 6. Conclusions

In conclusion, numerous inflammatory molecules and mechanisms are thought to be involved in the pathogenesis of obesity and obesity-related comorbidities. In the future, targeting inflammatory pathways might represent a necessary component of treatment of obesity to prevent life-threatening complications. For instance, glucagon-like peptide agonists are used in patients with obesity and obesity-associated comorbidities. Despite their effect on weight loss, these agents have also demonstrated anti-inflammatory capabilities [166,167,168]. Given the increasing prevalence of this metabolic condition, understanding the complex processes that drive the progression of adipose tissue inflammation might be crucial for preventing the development of metabolic, inflammatory, or even neoplastic complications. Importantly, obesity has been found to modulate cancer growth rates and the profiles of tumour microenvironments. However, the obesity complex should be further examined. Obesity also affects inflammatory diseases. For instance, in atopic dermatitis mouse models, the metabolic disorder increases the severity of the condition by altering the subtypes of immune cells that infiltrate cutaneous lesions [169]. In recent years, numerous studies have investigated the abilities of various drugs, natural compounds, and dietary elements to reduce adipose tissue inflammation [170]. Perhaps, future studies will investigate combinations that could suppress individual comorbidities of obesity, particular subtypes of cancer or inflammatory disorders, allowing for personalised treatment.
